# Intrathecal opioid versus ultrasound guided fascia iliaca plane block for analgesia after primary hip arthroplasty: study protocol for a randomised, blinded, noninferiority controlled trial

**DOI:** 10.1186/1745-6215-12-51

**Published:** 2011-02-21

**Authors:** Rachel J Kearns, Alan JR Macfarlane, Keith J Anderson, John Kinsella

**Affiliations:** 1 Glasgow University Academic Unit of Anaesthesia, Pain and Critical Care Medicine, Level 4, Walton Building, Glasgow Royal Infirmary, 91, Wishart Street, G31 2HT, Glasgow

## Abstract

**Background:**

Hip replacement surgery is increasingly common due to an ageing population, and rising levels of obesity. The provision of excellent pain relief with minimal side effects is important in order to facilitate patient mobilisation and rehabilitation.

Spinal opioids provide excellent analgesia but are associated with adverse effects. The fascia-iliaca block is an alternative technique which provides analgesia to the nerves innervating the hip. The success of fascia iliaca blocks has been demonstrated to be superior when using ultrasound compared to landmark techniques. However, the clinical benefit of this improvement has yet to be investigated.

The aim of this study is to compare the efficacy and safety of ultrasound guided fascia iliaca block with spinal morphine for hip replacement surgery.

**Methods/Design:**

This study is a randomised, blinded, placebo-controlled, noninferiority trial. Patients scheduled to undergo unilateral primary hip arthroplasty will receive a study information sheet and consent will be obtained in keeping with the Declaration of Helsinki. Patients will be randomised to receive either; (i) Ultrasound guided fascia iliaca block using levobupivacaine, plus spinal anaesthesia with hyperbaric bupivacaine containing no morphine, or (ii) sham ultrasound guided fascia iliaca block performed with sterile saline, and spinal anaesthesia containing hyperbaric bupivacaine and 0.1 mg of spinal morphine.

A total of 108 patients will be recruited. Primary outcome is post-operative morphine consumption in a 24 hour period. Secondary outcomes include; pain scores at 3, 6, 12, 24, 36 and 48 hours, episodes of respiratory depression, hypotension, nausea and vomiting, pruritus, sedation, time to first mobilisation and patient satisfaction.

**Conclusions:**

There are no studies to date comparing ultrasound guided fascia iliaca block with spinal morphine for pain control after hip arthroplasty. If the ultrasound guided fascia iliaca block provides pain relief which is not inferior to spinal morphine, then morphine could be removed from the spinal injection. This could reduce side effects and improve patient safety.

**Trial registration:**

This study has been approved by the West of Scotland Research Ethics Committee 4 (reference no. 10/S0704/43) and is registered with ClinicalTrials.gov (reference no. NCT01217294).

## Background

Hip surgery is increasingly being performed, often in elderly patients with significant co-morbidity [1]. Whilst the optimal anaesthetic technique is yet to be established [2], it is important that side effects are minimised to optimise patient safety and comfort and to facilitate rehabilitation. The main anaesthetic options are general anaesthesia (GA) and regional anaesthesia (RA) or a combination of the two. In a recent systematic review, regional anaesthesia (RA) was demonstrated to reduce post-operative pain, morphine consumption and nausea and vomiting compared to systemic analgesia [3].

Spinal anaesthesia is a RA technique commonly used in many patients undergoing hip arthroplasty [4]. Opioids are frequently added to the spinal anaesthetic in order to prolong and improve post-operative pain relief [5] and are associated with reduced post-operative morphine requirements in patients undergoing hip arthroplasty [6-8]. However, intrathecal opioids are associated with side effects including urinary retention, nausea and vomiting, pruritus and rarely, but most seriously, respiratory depression [9]. Such adverse effects can be uncomfortable for the patient, delay mobilisation, recovery and eventual discharge and occasionally be dangerous [10,11].

In patients undergoing hip arthroplasty peripheral nerve blockade has been shown to improve pain scores and reduce morphine consumption [3]. The fascia iliaca nerve block can provide sensory blockade of the main nerves which supply pain to the hip [12,13]. However, clinical success rates of this block when performed 'blindly' using traditional landmark techniques are variable [14]. Using ultrasound to locate nerves during peripheral nerve blockade has repeatedly been shown to increase success rates, reduce block onset time, increase block duration, reduce volumes of local anaesthetic required and increase patient satisfaction compared to traditional techniques [15-21]. The use of ultrasound guidance in the fascia iliaca plane block has been shown to increase success compared with the landmark technique [22]. In this study however, the clinical benefits of this increased success were not investigated.

Ultrasound guided fascia iliaca block has not yet been evaluated clinically as a method of providing post-operative analgesia following primary hip arthroplasty. We hypothesise that by increasing the success rate of the fascia iliaca block with ultrasound, it will be possible to achieve superior and more reliable analgesia than that obtained using the landmark based technique. The aim of this study is to assess whether the ultrasound guided fascia iliaca plane block can provide comparable post-operative analgesia to spinal morphine for primary hip arthroplasty. If this is the case, spinal opioid could be removed from the spinal anaesthetic. This could potentially reduce troublesome opioid related side effects and have significant safety benefits. The further investigation of this technique will provide a valuable contribution to existing knowledge, and could profoundly change current practice.

## Methods/Design

### Overview

This is a single centre, randomised, blinded, placebo-controlled, non-inferiority study [23]. This study has been approved by the West of Scotland Research Ethics Committee 4 (reference no. 10/S0704/43) and is registered with the ClinicalTrials.gov database (reference no. NCT01217294). This study will be performed in keeping with the requirements of the Declaration of Helsinki.

### Hypothesis

Ultrasound guided fascia iliaca plane block provides post-operative analgesia which is not inferior to that obtained with spinal morphine in patients undergoing primary hip arthroplasty.

### Objectives and Outcome Measures

This study aims to compare the efficacy and safety of ultrasound guided fasca iliaca block with intrathecal morphine in the provision of post-operative analgesia after primary hip arthroplasty. The primary outcome measure is post-operative morphine consumption in a 24 hour period as self administered using a patient controlled analgesia (PCA) pump. Secondary outcomes include pain scores at 3, 6, 12, 24, 36 and 48 hours, time to 1st morphine administration, episodes of respiratory depression, hypotension, nausea and vomiting scores, pruritus scores, sedation scores, urinary retention, time to first mobilisation and patient satisfaction.

### Study centre

Our centre is a tertiary referral facility for orthopaedics and trauma surgery with the necessary type and volume of clinical cases required for this study. There is a wealth of experience on the use of ultrasound guidance for regional anaesthetic techniques, including fascia iliaca block [22], within the department.

### Patients and enrolment

Patients scheduled to undergo unilateral primary hip arthroplasty will be invited to participate in the study during their routine pre-assessment visit two weeks prior to the date of surgery. Inclusion criteria are; ASA physical status I - III, 18 - 85 years of age, weight between 50 - 110 kg, and competence to consent. Exclusion criteria are; contraindications to fascia iliaca plane block or spinal anaesthesia such as coagulopathy, malignancy or infection in the inguinal area, preference for general anaesthesia, allergy to opioids, significant peripheral neuropathy or neurological disorder affecting the lower extremity, pregnancy, history of alcohol or drug dependency, history of long term strong opioid intake (i.e. WHO step 3 analgesics), and history of significant psychiatric conditions that may affect patient assessment.

All suitable patients will be given a patient information sheet approved by the West of Scotland Ethics Committee. They will be given an opportunity to review this before written informed consent is obtained prior to surgery.

### Consent

The process of consent will be in accordance with the Declaration of Helsinki. Patients will be fully informed that they are being asked to participate in a research study. The procedures involved in the study, and the chances of being assigned randomly to one of two groups will be explained in person and via an information sheet. A signed, consent form will be obtained from each patient and retained by the investigators. Patients will be made aware of their right to withdraw from the study at any time without adverse effects on their clinical care.

### Randomisation

A computer generated allocation sequence (in permuted blocks) will be created by an independent operator who is not directly involved with the study. Once created, the allocation sequence will be kept in a secure locked drawer making it inaccessible to all study personnel. Allocation concealment will be achieved using sequentially numbered sealed envelopes which are opaque when held to the light. When a patient is enrolled in the study, an administrator working within the Glasgow University Academic Unit of Anaesthesia will be contacted and asked to give the next numbered envelope to the anaesthetist who will make up the medications used in the study. The administrator will record the patient's details and the number of the envelope assigned to that patient. The allocation sequence will be accessed only when study data collection is complete or in any instance where unblinding of the study is thought to be essential in the provision of appropriate patient care.

Patients in the *Fascia Iliaca Group *will receive; spinal anaesthesia with hyperbaric bupivacaine at a dose between 10 and 15 mg, adjusted based on patient height and weight at the discretion of the attending anaesthetist, with no spinal morphine (0.1 ml sterile saline will be administered in its place). Ultrasound guided fascia iliaca block using 2 mg/kg levobupivacaine diluted to a total of 40 ml with sterile saline.

Post-operative analgesia will include Paracetamol 1 g four times daily and patient controlled analgesia (PCA) with morphine (1 mg bolus, 5 minute lockout period).

Patients in the *Spinal Morphine Group *will receive; spinal anaesthesia with hyperbaric bupivacaine as above, and with the addition of intrathecal morphine 100 micrograms (0.1 ml). "Sham" ultrasound guided fascia iliaca injection with 40 ml of sterile saline. Post-operative analgesia in the same manner as in the *Fascia Iliaca Group*.

### Blinding

The ultrasound guided fascia iliaca block will be performed by an anaesthetist deemed competent in this technique. The injectate solutions will have been pre-prepared in an aseptic manner by a separate anaesthetist not involved with post-operative data collection or analysis. In this way, the investigator performing the ultrasound guided fascia iliaca block and spinal injections will be blinded to the nature of the injectates. As patients will receive both spinal and ultrasound guided fascia iliaca injections, they will also be blinded. Data will be anonymised and collected by an investigator who is blinded to the patient's allocation.

### Intra-operative management

The anaesthetist looking after the patient in theatre will play no part in data analysis and will record the intra-operative proceedings as normal. The patient's participation in this study and the 2 possible anaesthetics that may have been administered will be documented on the anaesthetic chart. The randomisation code may be accessed if deemed necessary in the provision of optimal patient care. The patient may receive sedation if requested and as directed by the anaesthetic doctor. Fluid administration and the use of vasopressors will again be at the discretion of the anaesthetic doctor. All medications, with the exception of the medications used to perform the spinal or fascia iliaca block, will be detailed in the anaesthetic record. No anti-emetic will be administered peri-operatively unless specifically indicated.

### Postoperative management

After surgery, patients will be taken to the recovery room and monitored according to standard hospital policy. Pain will be treated, if required, with intravenous morphine every 5 - 10 min as directed by nursing staff. Patients will be familiarised with the Patient Controlled Analagesia (PCA) device and discharged once recovery room discharge criteria have been met. Patients will remain on oxygen for at least 24 hours and whilst receiving PCA morphine as is routine in our unit. Naloxone will be prescribed for sedation or respiratory depression as specified on the PCA protocol.

After a 48 hour period, data regarding pain scores, nausea, itch, sedation and hypotension will cease being collected as detailed in the primary and secondary outcomes. The investigator who collects the data will be blinded as to the nature of the anaesthetic administered. The time to first mobilisation will be assessed and the patient will continue to be monitored by physiotherapy staff until discharge. Any serious adverse events will prompt follow up. Patients will be seen routinely at 6 weeks following discharge by the arthroplasty specialist nurse. Symptoms of nerve damage will be actively sought at this consultation. Patients will be asked to rate their level of satisfaction with post-operative analgesia at both 48 hour and 6 week time points.

### Criteria for discontinuation

Every effort will be made to retain patients in the trial and to minimise withdrawals. Patients may request to be withdrawn from this study at any time. Intention to treat and "as treated" analyses will be performed.

### Data Collection

Data will be obtained from copies of the anaesthetic record, recovery room observation chart, PCA chart, ward observation chart and drug prescription chart. These charts will be reviewed after the first 48 hour post-operative period by a member of the research team. The researcher will be blinded to the anaesthetic technique used. All documentation relating to the study will be stored in an anonymised case report file unique to each patient. These case report files will be archived in a locked facility for a period of 10 years.

### Sample Size and Statistical Considerations

In the comparison of ultrasound guided fascia iliaca block with spinal opioid in patients undergoing primary hip replacement, we intend to compare an established technique in widespread practice (spinal morphine) with the less well investigated technique of ultrasound guided fascia iliaca block. The primary outcome of the study is 24 hour morphine consumption. This outcome is used commonly in trials of spinal opioid for hip arthroplasty surgery. Mean 24 hour morphine consumption after hip arthroplasty is reported to lie within the range 10 mg [6] to 30 mg [7] when using 0.1 mg intrathecal morphine. From our own audit data of patients receiving spinal opioid for hip arthroplasty over an 8 month period, mean 24 hour post-operative morphine consumption was 24.6 (SD 17.6) mg which lies within the reported range described above [6,7].

In order to calculate sample size, we used a method suggested for non-inferiority trials [23,24]. For this we made the following assumptions. Type 1 error (α) was set at 0.05; Type 2 error (β) at 0.8; and Z numbers based on one-tailed testing. We considered a difference between groups (δ*) of greater than 10 mg of morphine to be clinically significant. 10 mg of morphine equates to one subcutaneous dose of morphine commonly used in post-operative analgesia pain protocols [25].

The expected difference between the Control (spinal morphine) and Treatment (ultrasound guided fascia iliaca) groups (δ) is more difficult to estimate. To date, there is only one published trial looking at 24 hour post-operative morphine consumption after fascia iliaca block for hip arthroplasty, although this was performed with the landmark technique alone and did not employ ultrasound [13]. In this study, mean 24 hour post-operative morphine consumption in the fascia iliaca group was 23 mg. Therefore, there is a 1.6 mg difference between the mean 24 hour morphine consumption obtained from our audit data of patients receiving spinal morphine, and that obtained in a study looking at patients receiving fascia iliaca block for hip arthroplasty [13]. Thus, the number of patients required to adequately power this study is 108.

The null Hypothesis (H0) for this non-inferiority study is that the experimental treatment (ultrasound guided fascia iliaca block) is in fact inferior to the established treatment (spinal morphine) by more than the clinically significant amount (δ*). If H0 is rejected, the alternative hypothesis is that ultrasound guided fascia iliaca block is not inferior to IT opioid.

The study will be performed using both intention to treat and "as treated" analyses. In the intention to treat analysis, patients will be considered failures if they require general anaesthesia, or were unable to receive randomised treatment for any other reason. In the "as treated" analysis, only data from patients completing randomised treatment will be analysed.

Secondary data analyses will be carried out on all secondary outcomes. These will be compared between groups using t-test, and Mann-Whitney, or Chi-squared tests as appropriate.

It is anticipated that recruitment for this study will take between 12 and 18 months to complete if 1 to 2 patients are enrolled each week, using one surgeon to reduce surgical variability. Data collection for each patient will occur during the first 48 hours post-operatively and at a routine 6 week follow up appointment. No further follow up will be routinely arranged. Any patients requiring specific follow up will have this arranged on an individual basis.

We recognise that while this study is powered for the primary outcome, it is not powered for the secondary outcomes. However, the data we collect in this study will provide useful information for further studies looking specifically at these outcomes.

### Adverse Event Reporting and Safety

All adverse events will be recorded and discussed at monthly safety meetings by at least 2 investigators. If clinically indicated, the nature of the anaesthetic administered in the study may be revealed. After assessment by the Principle Investigator, any serious adverse events (SAEs) and suspected unexpected serious adverse reactions (SUSARs) will be reported to the Pharmacovigilance Office in the Robertson Centre for Biostatistics in Glasgow. All SUSARs will be reported to the Medicines and Healthcare products Regulatory Agency (MHRA) by the Pharmacovigilance Office at the Robertson Centre for Biostatistics in Glasgow.

## Discussion

### Risk Benefit Assessment

We expect that all patients will benefit from this study in view of the high level of post-operative monitoring and follow up which will be employed. In order to achieve blinding and improve the validity of the study, a "sham" ultrasound guided fascia iliaca block will be performed in patients in the *Spinal Morphine Group*. These patients will therefore receive an injection of an inactive substance (sterile saline) into the groin. As no local anaesthetic is being used in the sham block, potential risks will include; discomfort on injection, bleeding or bruising at the puncture site and nerve damage. Nerve damage is rare with fascia iliaca blocks as the needle is not directed towards the nerves themselves, but rather to lie in a plane between muscle layers. In the patients receiving fascia iliaca block with local anaesthetic, the risks are as before with the addition of local anaesthetic toxicity, although a pre-determined safe dose of local anaesthetic is being used.

Patients in the *Spinal Morphine Group *will receive intrathecal morphine. Spinal opioids have been used since 1979 to provide pain control after surgery [26]. Due to its widespread international use, intrathecal morphine has been extensively investigated in this setting and spinal morphine in combination with systemic morphine is a commonly used post-operative regime for many surgical procedures including hip arthroplasty [27-29]. Low dose intrathecal morphine can provide adequate analgesia whilst minimising side-effects [6-8]. Such side-effects include; delayed respiratory depression, pruritus, post-operative nausea and vomiting and urinary retention [5,30-32]. Although respiratory depression is rare with low doses of intrathecal morphine, [33] it is potentially life-threatening. Furthermore, the concomitant use of systemic opioids for post-operative analgesia may add to this risk. Previous research has concluded that 100 micrograms of intrathecal morphine combines analgesic efficacy whilst minimising the side effect profile [6,8]. Reassuringly, in a dose-finding study of intrathecal morphine for hip and knee surgery, there was no increased incidence of respiratory depression or hypoxaemia in patients receiving up to 0.3 mg of intrathecal morphine. This included elderly patients who had also received "significant doses of PCA morphine" [7].

A recent meta-analysis of 1300 patients was unable to define whether the use of intrathecal morphine increased the risk of respiratory depression [33]. Studies investigating the use of intrathecal opioid are generally not adequately powered to detect the incidence of respiratory depression. However, it is believed that lower doses result in a reduced risk [6-8,28,33,34]. A recent trial of 1915 patients receiving 0.15 mg of intrathecal morphine for Caesarean section found the incidence of a respiratory rate of less than 10 breaths per minute to be 0.26% and the need for naloxone 0.052% [34]. However, there is no evidence that there is an effective dose of intrathecal morphine that would completely preclude the occurrence of respiratory depression. An accurate estimate of the incidence of this complication would therefore require a trial containing very large numbers of patients and is impractical to undertake. In keeping with other investigators, we can not accurately predict the incidence of respiratory depression that may occur after the use of low dose intrathecal opioid and PCA morphine.

In the planning of this study, a number of measures have been employed to reduce this potential risk. These include; the utilisation of the lowest possible effective dose of intrathecal morphine (0.1 mg), the use of specific monitoring charts to ensure that the patient is monitored on an hourly basis, delivery of supplemental oxygen whilst receiving morphine via the PCA device, and the use of clear protocols to be followed by nursing staff in the management of adverse events. All nursing staff involved in post-operative patient care are competent and experienced in the management of patients who have received intrathecal and systemic morphine, and are trained in the necessary monitoring procedures.

Both spinal anaesthesia and peripheral nerve blockade are commonly performed for hip arthroplasty in the United Kingdom. Any possible risks must be weighed up against the risks of a general anaesthetic. Any adverse events relating to each of the procedures will be recorded by staff performing the study and any necessary investigations, treatment or follow up arranged thereafter.

## Competing interests

The authors declare that they have no competing interests.

## Authors' contributions

KA and AM conceived of the study. RK, AM, KA and JK designed the study. RK wrote the protocol. AM, KA and JK reviewed and optimised the protocol. RK has applied for funding for the study and is responsible for communicating with the Governing Bodies (West of Scotland Research Ethics Committee, NHS Research and Development and NHS Finance Departments). JK is the Principle Investigator for the study. All authors have read and approved of the final version of the manuscript.
